# Priority-setting for obesity prevention—The Assessing Cost-Effectiveness of obesity prevention policies in Australia (ACE-Obesity Policy) study

**DOI:** 10.1371/journal.pone.0234804

**Published:** 2020-06-19

**Authors:** Jaithri Ananthapavan, Gary Sacks, Vicki Brown, Marj Moodie, Phuong Nguyen, Lennert Veerman, Ana Maria Mantilla Herrera, Anita Lal, Anna Peeters, Rob Carter

**Affiliations:** 1 Deakin Health Economics, School of Health and Social Development, Institute for Health Transformation, Deakin University, Geelong, Australia; 2 Global Obesity Centre, School of Health and Social Development, Institute for Health Transformation, Deakin University, Geelong, Australia; 3 School of Medicine, Griffith University, Gold Coast, Australia; 4 Queensland Centre for Mental Health Research, Brisbane, Australia; 5 School of Public Health, The University of Queensland, Brisbane, Australia; 6 Institute for Health Metrics and Evaluation, University of Washington, Seattle, Washington, United States of America; Universitat Bremen, GERMANY

## Abstract

The aim of the ACE-Obesity Policy study was to assess the economic credentials of a suite of obesity prevention policies across multiple sectors and areas of governance for the Australian setting. The study aimed to place the cost-effectiveness results within a broad decision-making context by providing an assessment of the key considerations for policy implementation. The Assessing Cost-Effectiveness (ACE) approach to priority-setting was used. Systematic literature reviews were undertaken to assess the evidence of intervention effectiveness on body mass index and/or physical activity for selected interventions. A standardised evaluation framework was used to assess the cost-effectiveness of each intervention compared to a ‘no intervention’ comparator, from a limited societal perspective. A multi-state life table Markov cohort model was used to estimate the long-term health impacts (quantified as health adjusted life years (HALYs)) and health care cost-savings resulting from each intervention. In addition to the technical cost-effectiveness results, qualitative assessments of implementation considerations were undertaken. All 16 interventions evaluated were found to be cost-effective (using a willingness-to-pay threshold of AUD50,000 per HALY gained). Eleven interventions were dominant (health promoting and cost-saving). The incremental cost-effectiveness ratio for the non-dominant interventions ranged from AUD1,728 to 28,703 per HALY gained. Regulatory interventions tended to rank higher on their cost-effectiveness results, driven by lower implementation costs. However, the program-based policy interventions were generally based on higher quality evidence of intervention effectiveness. This comparative analysis of the economic credentials of obesity prevention policies for Australia indicates that there are a broad range of policies that are likely to be cost-effective, although policy options vary in strength of evidence for effectiveness, affordability, feasibility, acceptability to stakeholders, equity impact and sustainability. Implementation of these policies will require sustained co-ordination across jurisdictions and multiple government sectors in order to generate the predicted health benefits for the Australian population.

## Introduction

Globally, the obesity epidemic is firmly established with the prevalence of overweight and obesity nearly tripling over the last four decades [1, 2]. Australia has the fifth highest prevalence of obesity among countries in the Organisation for Economic Co-operation and Development (OECD) [3], with approximately 63% of the adult population and 27% of children experiencing either overweight or obesity [4]. In addition to the significant health burden associated with elevated body mass index (BMI) [5, 6], there is also a substantial economic impact on Australian society. Estimates for 2015 indicate approximately AUD3.8 billion is expended annually in direct medical costs and total costs of up to AUD8.6 billion when the indirect costs associated with lost productivity, government subsidies and forgone taxes were included [7].

There is global consensus that efforts to address obesity require a comprehensive societal response [8, 9]. This includes government policies across a wide range of sectors, such as health, education, agriculture, transport, trade and finance, as well as wide-scale action from the private sector and community groups [10]. Australian governments are demonstrating increased interest in addressing obesity with the announcement in late 2018 of plans to develop a National Obesity Strategy [11]. In order to inform evidence based decision-making, governments require not only evidence on the effectiveness of potential actions, but also their cost-effectiveness to prioritise the options for change that offer best value for money [12].

Over the last 15 years, the availability of economic evidence to guide decision-making on obesity-related policy has increased [13]. However, most evaluations have focused on medical treatments for obesity, such as pharmaceuticals and bariatric surgery, with limited evidence on the cost-effectiveness of obesity prevention policies [13]. Moreover, the evaluations have focused on single interventions, with different methods used in each study. Priority-setting decisions are likely to require comparative evidence on the cost-effectiveness of a suite of policy-relevant interventions that have been evaluated using consistent methods [13].

The Assessing Cost-Effectiveness (ACE) methodology was developed as a priority-setting tool to facilitate evidence-based decision-making. It combines technical rigour in the cost-effectiveness analyses with other considerations that influence policy decision-making [12, 14]. The ACE method has been used previously in two Australian obesity-related priority-setting studies: 'ACE-Obesity' [12, 15] and 'ACE-Prevention' [16]. A review of the economic credentials of the 22 obesity-related interventions from these Australian ACE studies showed that the majority of the interventions addressed the ‘downstream’ causes of and treatments for obesity, including medical interventions and interventions targeted at individual behaviour change [13]. Despite the increasing recognition that effective action on obesity will require strategies that change the obesogenic environment that we live in [17], there is limited comparative cost-effectiveness evidence of these ‘upstream’ policy interventions. However, the available evidence suggests that primary prevention interventions are more likely to be both health promoting and cost-saving (dominant interventions) than treatment interventions [13].

The aim of this priority-setting study was to focus on the current evidence gaps in the economic evidence by evaluating a range of ‘upstream’ obesity prevention policies (including both regulatory and program-based interventions) across multiple sectors and areas of governance (local, state and federal governments, and the private sector). In addition to the economic evidence, the study aimed to also provide a qualitative assessment of implementation considerations likely to be important to decision-makers [12].

## Methods

The ACE-Obesity Policy study was undertaken over the years 2012–2018. The research question was “What are the most effective, cost-effective, affordable and implementable policy options to prevent obesity across a range of settings?” This study used the ACE approach to priority setting, details of which have been previously published [12, 14, 16]; a brief description of its key features follows.

### The ACE approach to priority setting

The ACE approach acknowledges that decision-making requires not only technically rigorous cost-effectiveness analyses based on the best available evidence, but also consideration of the broader concerns of decision-makers and the range of issues that need to be deliberated when making policy decisions [12]. The approach involves a clearly specified and systematic process for intervention selection; the use of best available evidence for intervention effectiveness modelling; a standardised evaluation framework consistent with economic theory and concepts; placement of the cost-effectiveness results within the broader decision-making framework; and the involvement of stakeholders (project steering committee) throughout the priority-setting study.

### Intervention selection

Given the large number of heterogeneous policy options that could be considered for obesity prevention, we used the following intervention selection process. The overarching principles for selection were that the intervention should: i) be aimed at primary prevention rather than treatment, and ii) impact large population groups. Program-based interventions targeted at specific populations were required to be scalable for implementation across Australia. The interventions were identified in an iterative process. This involved an initial scan of relevant international and national authoritative reports related to obesity prevention, experience of the investigator team, and advice from the project steering committee made up of obesity researchers, epidemiologists, obesity advocates and policymakers. The list of potential interventions was reviewed annually to ensure all relevant interventions based on emerging evidence and global policy activity were considered for inclusion. Intervention selection was based on the potential impact of the intervention on overweight and obesity in Australia; the relevance of the intervention to current policy decision-making across Australian government jurisdictions and the private sector; and the availability of adequate evidence of intervention effectiveness to support the analyses. The selected interventions were mapped to a policy framework [10] to help ensure adequate representation of interventions across a range of sectors and levels of governance (local, state and federal government) and non-government decision-makers, such as the private sector. Interventions were classified as regulatory or program-based according to the most likely mechanism for policy implementation in the Australian setting (e.g. mandatory regulation, voluntary regulation, government guidelines, and national roll out of programs).

For interventions selected for inclusion, systematic searches of the grey and academic literature were undertaken to assess the strength of evidence for intervention effectiveness. Strength of evidence was assessed based on the quantity of evidence, study design and quality of the outcome measures used. The methods used for assessing strength of evidence are detailed elsewhere [18].

### Overview of cost-effectiveness methods

**Fig 1:**
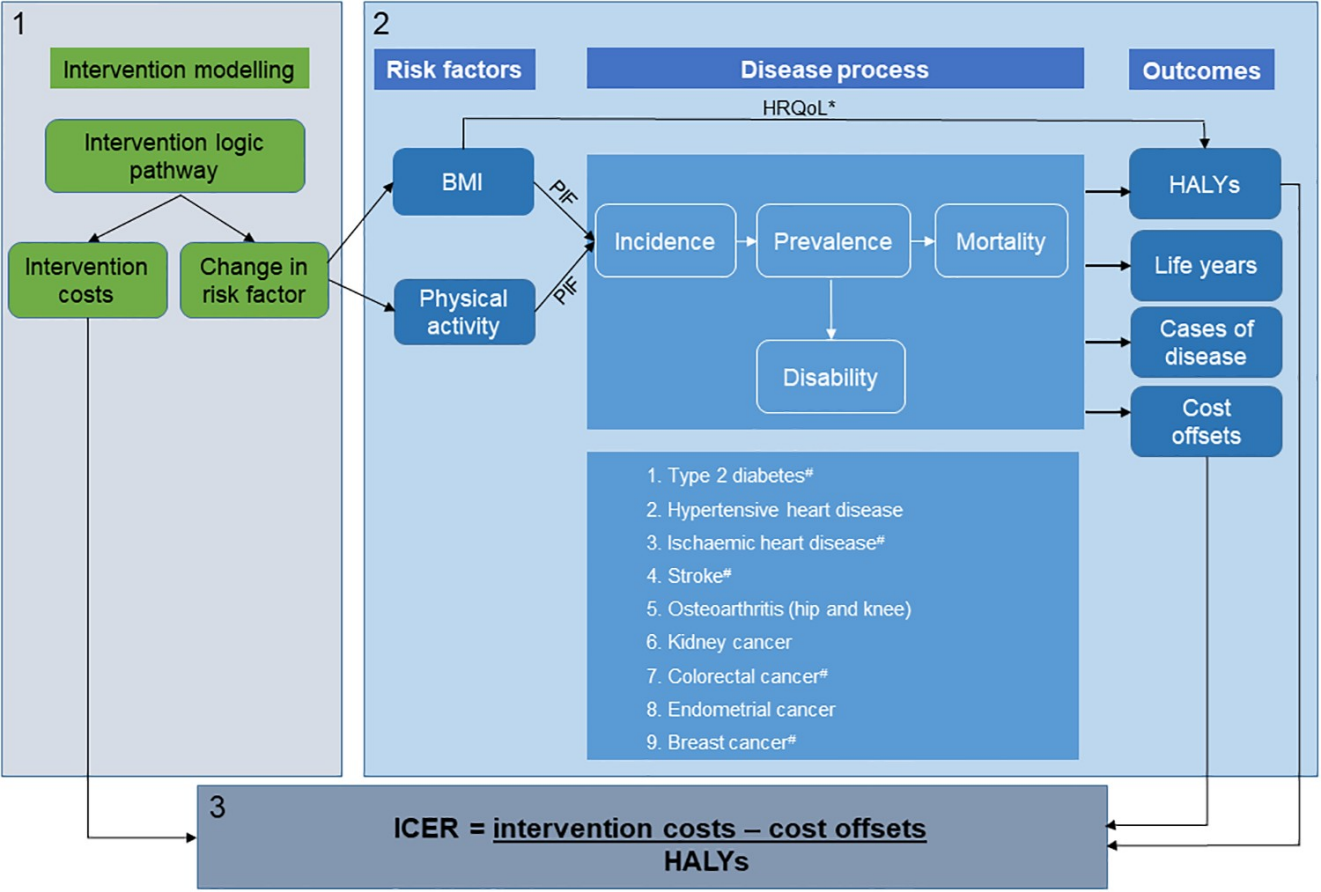
Schematic of the cost-effectiveness modelling process. BMI: body mass index; HALYs: health adjusted life years; HRQoL: health related quality of life; ICER: incremental cost-effectiveness ratio; PIF: potential impact fraction. * The health related quality of life related to BMI status in children, independent of disease status is incorporated into the HALYs ^#^ Diseases causally related to physical inactivity risk factor.

Once interventions were selected and prioritised for evaluation, modelling the cost-effectiveness consisted of three steps (Fig 1). The first step involved developing logic pathways to identify the relevant actions required for the intervention to have an impact on population BMI and physical inactivity levels when implemented in the Australian context. The logic pathways were used to model the intervention costs and the impact of the intervention on one or both risk factors. The second step involved using the ACE-Obesity Policy model (described below) to estimate the impact of changes in the population prevalence of risk factors on long-term health outcomes and healthcare costs. The third step involved aggregating the incremental costs and benefits of the intervention compared to a no intervention comparator to estimate the incremental cost-effectiveness ratio (ICER). An intervention was considered cost-effective if the ICER was less than AUD50,000 per health adjusted life year (HALY) gained. The willingness-to-pay threshold for health gains in Australia is not explicit, however previous ACE studies have used a threshold of AUD50,000 per HALY gained which is consistent with previous funding decisions made by the Australian Pharmaceutical Benefits Advisory Committee (PBAC) [19].

### Intervention costs

This study used a limited societal perspective and therefore costs accruing to various government sectors, private companies and individuals were included in the analyses. Cost components varied by intervention, however the main elements included recruitment costs for targeted interventions, cost of passing legislation for mandatory policy interventions and the key elements of intervention implementation, administration, compliance assessment and maintenance over the duration of the intervention. The cost to industry of implementing the intervention was included in the analyses, with impacts on company revenue included in sensitivity analyses when adequate data were available. Deadweight losses associated with taxation policies were included in sensitivity analyses, however potential overall welfare losses to individuals were not included. Time and travel costs accrued by individuals were included, however time costs of children were excluded. Other excluded costs were productivity costs, and intervention research, development and evaluation costs (interventions were assumed to be in steady state and running at their full effectiveness potential).

Unit costs were collected in Australian dollars (AUD) for the 2010 reference year largely from administrative databases. Wages included salary on-costs (13% for public sector and 14% for private sector) and 17.5% leave loading. Time costs were valued at gender free wage rates [20]. A detailed costing study [21] was undertaken to estimate the cost of passing legislation in the Australian Federal Parliament and was used for all interventions requiring legislation. All costs were adjusted to 2010 values using the Health Price Index for healthcare related costs and the Gross Domestic Product index for non-healthcare related costs [22].

### Impact of intervention on risk factors

Interventions that impacted food consumption were modelled using food composition data from the Food Standards Australia New Zealand NUTTAB 2010 database [23] and The George Institute Food Composition database [24]. Changes in kilojoule (kJ) intake resulting from the intervention were converted to corresponding changes in weight in kilograms (kg) using validated energy balance equations for adults [25] and children [26]. Weight changes were converted to changes in BMI using the average height by age and sex for those aged 18 years and over. In children, BMI z-scores were converted to changes in BMI using World Health Organization (WHO) standardised growth charts [27].

Interventions impacting physical activity were assumed to have an impact not only on physical activity levels, but also BMI based on the assumption that the increased energy expenditure was not compensated by increased energy intake. Changes in physical activity levels were measured in metabolic equivalent task (MET) minutes per week [28, 29] and converted to a kJ deficit [28] resulting in changes in BMI.

To allow comparability across interventions included in this priority-setting study, we assumed that the impact of regulatory interventions on risk factors were maintained over the lifetime of the modelled population. However, individual intervention evaluations tested the impact of this assumption with variations in maintenance of effect tested in scenario analyses. The duration of intervention effect varied for program-based interventions according to the characteristics of each intervention.

### The ACE-Obesity Policy model

A proportional, multi-state life table Markov cohort model was developed to quantify the relationship between characteristics of the population, risk factor prevalence and obesity related diseases to estimate the longer-term outcomes of obesity prevention initiatives. The Markov modelling approach is widely used in economic evaluations and can adequately capture the epidemiology and associated costs of chronic diseases associated with obesity [30]. Details of the model have been previously published [31] and are described here briefly.

The model simulates the 2010 Australian population (aged 2–100 years) and estimates the effect of intervention-related changes in one or more risk factors on the incidence of diseases related to the relevant risk factors over the lifetime of the population. Over time, reduced incidence of disease results in reductions in prevalence and mortality compared to no intervention, thereby producing long-term health benefits and healthcare cost-savings.

Potential impact fractions (PIFs) were calculated using relative risks (RR) from the Global Burden of Disease study [32] and were used to calculate the change in disease incidence resulting from the intervention. PIFs were calculated using the distribution shift method for BMI [33] and the relative risk shift method for physical activity [34]. Interventions targeting sedentary behaviour were modelled based on changes in METs, however the benefits of reduced sedentary behaviour independent of physical activity and BMI were not modelled.

The model included nine diseases (kidney cancer, colorectal cancer, endometrial cancer, breast cancer, type 2 diabetes, hypertensive heart disease, ischaemic heart disease, stroke, and osteoarthritis of the hip and knee). All diseases were causally related to BMI and five were related to physical activity (colorectal cancer, breast cancer, type 2 diabetes, ischaemic heart disease, and stroke). The joint effects of BMI and physical inactivity on common diseases were accounted for by adjusting the relevant RRs using a multiplicative function [35]. Disease-specific lifetables were modelled for each disease to capture the morbidity and mortality impacts. Disability weights from the Global Burden of Disease study [36], which provides a common source and consistent methods for the valuation of health states, were used to calculate the morbidity impacts.

### Cost-effectiveness modelling

The primary health outcome was the incremental HALYs gained from the intervention. HALYs were calculated to incorporate the population level changes in the morbidity and mortality associated with the nine diseases included in the model (which only impact adults in the model) and the health related quality of life impact of elevated BMI in childhood [37]. Unit costs for the included diseases were provided to us by the Australian Institute of Health and Welfare for a previous study [16] and were inflated from 2001 values to 2010 values to estimate the healthcare costs-savings resulting from the intervention. All future costs and benefits were adjusted to 2010 values using a 3% discount rate as used in previous Australian priority-setting studies [12, 16]. The net costs of the intervention were divided by the net HALYs to calculate the ICER.

The modelling was undertaken using Microsoft Excel 2013 software. Monte Carlo simulation using the Excel add-in software Ersatz [38] was used to incorporate parameter uncertainty in the model. Two thousand iterations of the model with varying parameter values defined by the most likely distribution were used to present uncertainty around the outputs (with 95% uncertainty intervals (UI)). Various univariate sensitivity analyses were undertaken to test the key assumptions related to specific interventions. For parameters with large uncertainty and large impacts on the ICER results, threshold analyses were undertaken to inform the threshold value at which the intervention would remain cost-effective (i.e. have an ICER below AUD50,000 per HALY gained).

The key reporting items based on the Consolidated Health Economic Evaluation Reporting Standards (CHEERS) checklist [39] are reported in S1 Table.

### Implementation considerations

The cost-effectiveness results were placed within a broader decision-making framework by assessing various factors that were considered by the project steering committee to be of importance when funding and policy implementation decisions are made. The six factors considered were strength of evidence for intervention effect, equity impacts, acceptability of the intervention to various stakeholders, feasibility of intervention implementation and sustainability of the intervention once implemented (see Table 1). The assessment of each of the implementation considerations was informed by the literature, program logic, real-world evidence and parallel evidence from other relevant policy contexts. The impact of the intervention on these factors were assessed as high, medium or low, or positive, negative or neutral. Final judgements were based on a deliberative process where the project team assessed all forms of evidence and assessed the interventions relative to the other interventions in this study to ensure internally consistent ratings.

**Table 1:** Implementation considerations.

Implementation consideration	Key considerations	Assessment
Strength of evidence	Based on the evidence framework and classified into certainty of effect on BMI outcomes.	Low
		Medium
		High
	When BMI outcomes were not available, classification was based on certainty of effect on physical activity or dietary outcomes.	Low
		Medium
		High
Equity	Composite definition that considered both process and outcome dimensions of equity:	Negative
	Impact on the equity of distribution of disease or health status, access to or utilisation of specific interventions. Out-of-pocket costs relative to income.	Neutral
		Positive
Acceptability	Acceptability to the general public.	Low
		Medium
		High
	Acceptability to government.	Low
		Medium
		High
	Acceptability to industry.	Low
		Medium
		High
Feasibility	Feasibility of implementation based on local/national/international experience in implementing similar policy interventions.	Low
		Medium
		High
Sustainability	Asks the question how sustainable is the intervention after implementation. Considers the mechanism of intervention. Mandatory regulation was assessed as more sustainable than scale up of program based interventions. Other considerations included the level of ongoing funding required for sustained implementation and the likelihood that the intervention would result in sustained behaviour change.	Low
		Medium
		High

### Presentation of results

The results of the cost-effectiveness analysis are presented in a technical results league table with the interventions ranked by their base case ICER. Dominant interventions (resulting in both cost-savings and health gains) do not have interpretable ICERs and therefore were ranked by total HALYs gained. To provide an indication of the short-term budget impact, the intervention costs over the first three years of implementation are also reported. Although the effect size and the variability around it were incorporated into the technical cost-effectiveness results and uncertainty intervals, we also provide a comparative assessment of the strength of evidence of the intervention on BMI outcomes, given the importance of the evidence base in policy-making [40].

A comparative assessment of the evaluated interventions on the implementation considerations is presented in a league table, ordered by the strength of evidence for BMI outcomes, then by strength of evidence on nutrition or physical activity outcomes, followed by equity considerations. The final sorting was based on the number of ‘positive’ or ‘high’ ratings. To place these results within the context of the cost-effectiveness results, the base case ICER and where the intervention ranks in the cost-effectiveness league table is also presented.

## Results

Economic evaluations were completed for 16 obesity prevention interventions. Table 2 provides a brief definition of each intervention including the type of intervention (regulatory or program-based), key modelling specifications (target population, risk factors addressed and the duration of intervention implementation and effect maintenance) and details of the likely sectors, industries and government jurisdictions involved in the policy decision, funding and implementation of the intervention. Thirteen of the 16 interventions were multi-sectoral, requiring the involvement of more than one sector or government department. All 16 interventions require input from the federal government, with state government involvement required for ten interventions. Seven interventions targeted the whole modelled population (aged 2–100 years), four targeted children and five targeted the adult or working age population. Ten interventions targeted population nutrition and addressed BMI as the key risk factor. Two of the four physical activity interventions focused on sedentary behaviour. The two multi-component programmatic interventions (‘Community–based interventions’ and ‘Financial incentives for weight loss provided by private health insurers’) impacted both physical activity and nutrition, however the evidence of effectiveness was limited to BMI outcomes and therefore only the intervention’s impact on BMI was modelled. A mix of regulatory (9/16) and program-based interventions (7/16) were evaluated.

**Table 2:** Interventions included in the ACE-Obesity Policy study.

Intervention and classification	Intervention description	Model specifications (target population, risk factors modelled, and duration of intervention/ effect maintenance)	Government Sector	Industry involved/ impacted	Jurisdiction for intervention implementation
Alcohol price increase: uniform volumetric tax [41] (regulatory)	Mandatory legislation to replace the current alcohol taxation system with a uniform volumetric tax equal to AUD1.07 per standard drink, applied across all alcohol products.	14–100 year olds BMI Lifetime/lifetime	Multi-sectoral (Health, Industry, Treasury)	Alcohol producers, suppliers and retailers Bars and restaurants	Federal and state governments
Community–based interventions [31] (program)	Co-ordinated program of community-level strategies to promote healthy eating and physical activity. Effectiveness limited to children.	5–18 year olds BMI 3 years/lifetime	Multi-sectoral (across all local government sectors)	Local businesses	Local government Likely to require funding from state/federal government
Financial incentives for weight loss provided by private health insurers (program)	AUD200 cash payment per year for five years contingent on meeting weight loss/maintenance goals alongside a one year commercial weight loss program. Eligibility limited to people with overweight/obesity, who have private health insurance with extras cover.	18–100 year olds BMI 5 years/11 years	Health	Private health insurers	Federal government
Fuel excise: 10c per litre increase [42] Regulatory	Mandatory legislation to increase the existing national fuel excise tax by AUD0.10 per litre.	18–64 year olds BMI/PA/Injury Lifetime/lifetime	Multi-sectoral (Transport, Treasury, Regional Affairs)	Fuel producers and importers	Federal government
Menu kilojoule labelling on fast food (regulatory)	Mandatory legislation for fast food outlets to display energy content of foods and drinks on menus accompanied by a government sponsored education campaign.	2–100 year olds BMI Lifetime/lifetime	Health	Fast food	Predominantly state governments with input from the federal government
National mass media campaign related to sugar-sweetened beverages (program)	Three-year national mass media campaign (12 six-week waves) to encourage reduced consumption of sugar sweetened beverages.	18–100 year olds BMI 3 years/3 years	Health	Media	Federal government
Package size cap on sugar-sweetened beverages [43] (regulatory)	Mandatory legislation to restrict the manufacturing of single-serve sugar-sweetened beverages (carbonated drinks) over 375ml.	2–100 year olds BMI Lifetime/lifetime	Multi-sectoral (Health, Industry)	Beverage manufacturers	Federal government
Reformulation in response to the Health Star Rating (HSR) system [44] (regulatory)	Impact of the government-endorsed voluntary HSR system on product reformulation.	2–100 year olds BMI Lifetime/lifetime	Multi-sectoral (Health, Industry)	Food and beverage manufacturers	Federal and state governments
Reformulation to reduce sugar in sugar-sweetened beverages [43] (regulatory)	Setting of voluntary targets for manufacturers to reduce the sugar content of sugar-sweetened beverages.	2–100 year olds BMI Lifetime/lifetime	Multi-sectoral (Health, Industry)	Beverage manufacturers	Federal and state governments
Restricting television advertising of unhealthy foods [45] (regulatory)	Mandatory legislation restricting unhealthy food and beverage marketing on free to air television until 9.30pm.	5–15 year olds BMI Lifetime/lifetime	Multi-sectoral (Health, Communications)	Broadcasters, media and advertising	Federal government
Restrictions on price promotions of sugar- sweetened beverages [46] (regulatory)	Mandatory legislation restricting the price promotion, temporary price discounts, and multi-buy specials of sugar-sweetened beverages (sugar-sweetened carbonated drinks, flavoured water, sports, energy, and fruit drinks; and cordials (concentrates) containing added sugar).	2–100 year olds BMI Lifetime/lifetime	Multi-sectoral (Health, Industry)	Supermarkets, other retailers	Federal and state government
School-based intervention to reduce sedentary behaviour (program)	Based on the Transform-Us! program [47]. Education and behaviour change techniques (e.g. standing lessons) and environmental strategies implemented by teachers in primary schools.	8–9 year olds BMI/PA Lifetime/lifetime	Multi-sectoral (Health, Education)	None	Federal and state government
School-based intervention to increase physical activity (program)	Based on the Transform-Us! program [47]. Education and behaviour change techniques (e.g. active breaks) and environmental strategies (e.g. active play equipment) implemented by teachers in primary schools.	8–9 year olds BMI/PA Lifetime/lifetime	Multi-sectoral (Health, Education)	None	Federal and state government
Sugar-sweetened beverages tax– 20% [21] (regulatory)	20% sales tax applied to sugar-sweetened beverages (sugar-sweetened carbonated drinks, flavoured water, sports, energy, and fruit drinks, and cordial concentrates containing added sugar).	2–100 year olds BMI Lifetime/lifetime	Multi-sectoral (Health, Industry, Treasury)	Beverage manufacturers	Federal government
Supermarket shelf tags on healthier products (program)	Voluntary intervention to encourage and assist supermarket chains to install and maintain shelf tags to alert customers to healthier products (4.5 and 5 HSR products).	2–100 year olds BMI 3 years/3 years	Multi-sectoral (Health, Industry)	Supermarkets	Predominantly state governments with input from the federal government
Workplace intervention to reduce sedentary behaviour [48] (program)	Multi-component workplace-delivered intervention (information, standing desks, individual health coaching) to reduce sedentary behaviour and increase physical activity.	18–65 year olds PA 1 year/5 years	Multi-sectoral (Health, Industry)	Desk work based workplaces	Federal and state government

BMI: body mass index; HSR: Health Star Rating; PA: physical activity

Table 3 shows a league table of the technical cost-effectiveness results. All 16 interventions were cost-effective. Eleven interventions were assessed as ‘dominant’, where the intervention resulted in health gains and net cost-savings over the lifetime of the modelled population. The five non-dominant interventions at the bottom of the league table had mean ICERs ranging from AUD1,728 to 28,703 per HALY gained. The intervention modelling predicted significant health gains resulting from implementation of the interventions with the middle 50% of values ranging from 28,981 to 73,883 total HALYs gained. There were outliers on both sides with ‘Alcohol price increase: uniform volumetric tax’ predicted to produce over 471,000 total HALYs gained and ‘Fuel excise: 10c per litre increase’ resulting in an estimate of 237 HALYs gained. Total intervention costs ranged markedly from AUD4.4 million for ‘Fuel excise: 10c per litre increase’ to AUD1.7 billion for ‘Financial incentives for weight loss provided by private health insurers’. The middle 50% of values for total intervention costs ranged from AUD15 million to AUD170 million. The intervention costs were mostly concentrated in the first three years of implementation. Total net costs ranged from–AUD4.8 billion (cost savings) for ‘Alcohol price increase: uniform volumetric tax’ to over AUD1 billion for ‘Financial incentives for weight loss by private health insurers’. The middle 50% of values for total net costs ranged from–AUD638 million (cost savings) to AUD2 million. The three interventions at the top of the league table (‘Alcohol price increase: uniform volumetric tax’, ‘Sugar-sweetened beverages tax– 20%’ and ‘Restricting television advertising of unhealthy foods’) were regulatory interventions addressing population nutrition. Seven of the eleven dominant interventions and six of the top eight interventions were regulatory interventions.

**Table 3:** Cost-effectiveness results league table.

Intervention	ICER (95% UI)	Total HALYs gained (95% UI)	Total intervention costs (95% UI)	Intervention costs in the first 3 years	Total healthcare cost offsets (95% UI)	Total net cost (95% UI)*	Strength of evidence BMI
Alcohol price increase: uniform volumetric tax	Dominant (Dominant to Dominant)	471,165 (413,231 to 535,804)	$32M ($31M to $33M)	$25M	-$4.8B (-$5.5B to -$4.3B)	-$4.8B (-$5.5B to -$4.2B)	Low
Sugar-sweetened beverages tax—20%	Dominant (Dominant to Dominant)	175,300 (68,700 to 277,800)	$120M ($92M to $162M)	$12M	-$1.7B (-$2.7B to -$650M)	-$1.6B (-$1.9B to -$1.5B)	Low
Restricting television advertising of unhealthy foods	Dominant (Dominant to Dominant)	88,396 (54,559 to 123,199)	$6M ($6M to $7M)	$1.5M	-$784M (-$1.0B to -$376M)	-$778M (-$1.0B to -370M)	Low
Package size cap on sugar-sweetened beverages	Dominant (Dominant to Dominant)	73,883 (57,038 to 96,264)	$210M ($148M to $273M)	$144M	-$751M (-$991M to -$556M)	-$541M (-$793M to -$341M)	Low
Supermarket shelf tags on healthier products	Dominant (Dominant to Dominant)	72,532 (31,857 to 116,010)	$9M ($7M to $12M)	$9M	-$647M (-$1.0B to -$290M)	-$638M (-$1.0B to -$282M)	Low
Menu kilojoule labelling on fast food	Dominant (Dominant to Dominant)	63,492 (37,540 to 107,253)	$170M ($131M to $209M)	$37M	-$672M (-$1.2B to -$368M)	-$502M (-$1.0B to -$191M)	Low
School-based intervention to reduce sedentary behaviour	Dominant (Dominant to Dominant)	61,989 (15,834 to 107,779)	$15M ($10M to $25M)	$14M	-$661M (-$1.1B to -$173M)	-$646M (-$1.1B to -$155M)	Medium
School-based intervention to increase physical activity	Dominant (Dominant to Dominant)	60,780 (15,007 to 109,413)	$10M ($7M to $15M)	$10M	-$641M ($1.1B to -$165M)	-$631M (-$1.1B to -$155M)	Medium
Restrictions on price promotions of sugar-sweetened beverages	Dominant (Dominant to Dominant)	48,336 (36,293 to 63,932)	$17M ($10M to $26M)	$5M	-$498M (-$653M to -$378M)	-$481M (-$638M to -$361M)	Low
Reformulation to reduce sugar in sugar-sweetened beverages	Dominant (Dominant to Dominant)	28,981 (21,884 to 37,976)	$45M ($31M to $58M)	$31M	-$295M (-$391M to -$217M)	-$251M (-$347M to -$217M)	Low
National mass media campaign related to sugar-sweetened beverages	Dominant (Dominant to Dominant)	13,958 (11,946 to 16,319)	$31M ($28M to $33M)	$31M	-$157M (-$178M to -$137M)	-$127M (-$148M to -$106M)	Low
Reformulation in response to the Health Star Rating system	$1,728 (Dominant to 10,445)	4,207 (2,438 to 6,081)	$46M ($32M to $60M)	$31M	-$42M (-$62M to -$22M)	$5M (-$21M to $28M)	Low
Financial incentives for weight loss provided by private health insurers	$7,376 ($1,022 to $15,146)	140,110 (112,899 to 170,243)	$1.7B ($882M to $2.7B)	$1.6B	-$692M (-$890M to -$515M)	$1.0B ($157M to $2.0B)	High
Fuel excise: 10c per litre increase	$7,684 ($7,617 to $10,919)	237 (138 to 351)	$4M ($3M to $5M)	$4M	-$2M (-$4M to -$1M)	$2M ($1M to $3M)	Low
Community-based interventions	$8,155 ($237 to $81,021)	51,792 (6,816 to 96,972)	$878M ($794M to $963M)	$878M	-$452M (-$854M to -$58M)	$426M ($3M to $823M)	High
Workplace intervention to reduce sedentary behaviour	$28,703 ($24,547 to $34,088)	7,492 (6,555 to 8,428)	$269M	$269M	-$54M (-$63M to -$46M)	$215M ($207M to $224M)	Low

B: billions; BMI: body mass index; HALYs: health adjusted life years; ICER: incremental cost-effectiveness ratio; M: million; UI: uncertainty interval; $ Australian dollars 2010.

The willingness-to-pay threshold for this analysis is $50,000 per health adjusted life year gained. Dominant: the intervention is both cost-saving and improves health. Negative numbers indicate cost saving.

*Due to rounding, the total net costs may differ slightly from the difference between the total intervention cost and the healthcare cost offsets.

Table 4 shows the interventions ranked based on the implementation considerations. The top four interventions were program-based interventions, with two scoring high and two medium on the strength of evidence on BMI outcomes. Twelve interventions scored either high or medium on one of the two strength of evidence categories. Twelve interventions were assessed as having a neutral or positive impact on equity. ‘Financial incentives for weight loss provided by private health insurers’ was assessed as having a negative impact on equity of access because the eligible population was restricted to those who could afford private health insurance cover. The other three negative equity impact assessments (‘Alcohol price increase: uniform volumetric tax’, ‘Fuel excise: 10c per litre increase’ and ‘Restrictions on price promotions of sugar-sweetened beverages’) were based on the intervention impacting the price of regulated products and therefore affecting lower income groups disproportionately. Eight interventions were assessed as being highly acceptable to the public. Public acceptability corresponded well with government acceptability for the majority (12/16) of interventions. Low public acceptability was related to interventions that reduced the ‘value for money’ of products, and corresponded well with low acceptability to industry. Feasibility and sustainability of the interventions was generally favourable. The two interventions that scored a ‘low’ rating for feasibility lacked evidence of effective implementation in any context (‘Package size cap on sugar-sweetened beverages’ and ‘Restrictions on price promotions of sugar- sweetened beverages’). ‘Workplace intervention to reduce sedentary behaviour’ scored low on sustainability as sustained behaviour change from this intervention would require ongoing funding for standing desk repairs and replacement and continued employee education.

**Table 4:** Implementation considerations league table.

Intervention	Strength of evidence BMI	Strength of evidence PA/diet	Equity	Acceptability Public	Acceptability Government	Acceptability Industry	Feasibility	Sustainability	ICER {cost-effectiveness ranking from Table 3}
Community-based interventions	High	N/A	Neutral	High	High	High	Medium	Medium	$8,155 {15}
Financial incentives for weight loss provided by private health insurers	High	N/A	Negative	Medium	High	Medium	High	Medium	$7,896 {13}
School-based intervention to reduce sedentary behaviour	Medium	Medium	Positive	High	High	High	High	Medium	Dominant {7}
School-based intervention to increase physical activity	Medium	Medium	Positive	High	High	High	High	Medium	Dominant {8}
Reformulation in response to the Health Star Rating system	Low	Medium	Positive	High	High	Medium	High	Medium	$1,728 {12}
Restricting television advertising of unhealthy foods	Low	Medium	Positive	High	Medium	Low	High	High	Dominant {3}
Reformulation to reduce sugar in sugar-sweetened beverages	Low	Medium	Positive	Medium	High	Medium	High	Medium	Dominant {10}
Menu kilojoule labelling on fast food	Low	Medium	Neutral	High	High	Medium	High	High	Dominant {6}
Supermarket shelf tags on healthier products	Low	Medium	Neutral	High	High	Medium	High	Medium	Dominant {5}
Workplace intervention to reduce sedentary behaviour	Low	Medium	Neutral	High	High	Medium	Medium	Low	$28,703 {16}
Sugar-sweetened beverages tax—20%	Low	Medium	Neutral	Medium	Medium	Low	High	High	Dominant {2}
Alcohol price increase: uniform volumetric tax	Low	Medium	Negative	Low	Medium	Low	High	High	Dominant {1}
Package size cap on sugar-sweetened beverages	Low	Low	Positive	Low	Low	Low	Low	Medium	Dominant {4}
National mass media campaign related to sugar-sweetened beverages	Low	Low	Neutral	Medium	Medium	Medium	High	Medium	Dominant {11}
Fuel excise: 10c per litre increase	Low	Low	Negative	Low	Low	Medium	High	High	$7,684 {14}
Restrictions on price promotions of sugar-sweetened beverages	Low	Low	Negative	Low	Low	Low	Low	High	Dominant {9}

BMI: body mass index; ICER: incremental cost-effectiveness ratio; PA: physical activity; The willingness-to-pay threshold for this analysis is $50,000 per health adjusted life year gained. Dominant: the intervention is both cost-saving and improves health. Strength of evidence BMI relates to evidence for the effect of the intervention on body mass index. Strength of evidence PA/diet relates to evidence for the effect of the intervention on physical activity or dietary outcomes (intervention dependent).

## Discussion

All 16 interventions evaluated in this priority-setting study were assessed as being cost-effective approaches to addressing obesity in the Australian population. The modelling predicted substantial health gains over the lifetime of the modelled population. Intervention costs were largely accrued during the initial years of the intervention, with healthcare cost savings accruing over varying time horizons.

The majority of the interventions evaluated (11/16) were predicted to result in long-term cost-savings and health gains. Dominance was largely driven by the relatively low cost of the dominant interventions (mean intervention costs for the non-dominant interventions were nine-fold larger than the dominant interventions, whereas the mean health gains for the dominant interventions were less than three times larger than the non-dominant interventions). The dominant interventions also tended to be universal, non-targeted interventions impacting large populations.

The three most cost-effective interventions (‘Alcohol price increase: uniform volumetric tax’ [41], ‘Sugar-sweetened beverages tax– 20%’ [21], and ‘Restricting television advertising of unhealthy foods’ [45]) were all regulatory interventions. The regulatory interventions appeared higher on the cost-effectiveness league table compared to program-based interventions. This result was driven by the higher costs of program-based interventions (intervention costs for program-based interventions were over 12 fold greater than regulatory interventions, however the health gains were less than two times larger for the regulatory intervention). Evaluations of program-based interventions were facilitated by more detailed costing data, and therefore the availability of costing data and differential costing methodology may have influenced this finding. Although we attempted to account for industry impacts in the evaluations, limited data availability meant that the impact on industry revenue resulting from regulatory interventions was not captured in the base case results, potentially underestimating the true cost of regulatory interventions. As part of the evaluation of one intervention (‘Restricting television advertising of unhealthy foods’ [45]), we conducted sensitivity analyses to include the impact of short term revenue losses to industry (television broadcasters) on cost-effectiveness results, and found that the intervention remained ‘dominant’. For another intervention (‘Restrictions on price promotions of sugar-sweetened beverages’ [46]), we conducted a threshold analysis to determine the level of industry response at which the intervention would no longer be cost-effective. As evidence of the impacts on industry and the various industry responses to obesity prevention intervention emerges, studies should aim to better incorporate these into cost-effectiveness analyses.

The results of this priority-setting study are consistent with other obesity-related studies both in Australia [12, 16] and internationally [49–51]. These studies have consistently found that policy-based interventions targeting the food environment, and regulatory interventions that are relatively low cost and have high population reach are most cost-effective.

Interventions targeted at children were modelled under the assumption that the BMI change resulting from the intervention was maintained over the lifetime of the affected population. Given that governments are particularly interested in protecting children and therefore acting on childhood obesity [8, 52], it will be important to test this assumption which requires further research to explore the maintenance of obesity prevention intervention effects over time. Future research investigating intervention characteristics that facilitate the durability of effects is also required.

Despite all interventions having a strong program logic of how they would impact on obesity levels, there was limited empirical evidence of the effectiveness of most interventions on BMI outcomes. Only four interventions scored high or medium on BMI outcomes; all were programmatic in nature. There are several explanations for these findings. Firstly, the regulatory interventions are not conducive to being evaluated using high quality randomised controlled trials [53, 54]. Secondly, the lack of effective implementation of promising interventions both in Australia and internationally means there is a lack of real-world evidence of intervention effect [55]. And finally, even if implemented, most interventions have not been followed up long enough to detect impacts on BMI. It is important to note that the majority of the interventions (12/16) demonstrated adequate evidence of impact on BMI, dietary or physical activity outcomes (scored medium or higher). For these interventions to impact on BMI, the key assumption relates to the extent of compensatory behaviour (e.g. compensatory eating or physical inactivity/sedentariness following a change in one aspect of diet or physical activity). Future research should allow for longer-term follow up and focus on the nature and extent of compensatory behaviour.

Given the obesity gradient in Australia where persons of lower socio-economic position have higher levels of overweight and obesity [4], action on obesity is likely to have a positive impact on the equity of health outcomes. This is reflected in the majority of the interventions (12/16) being assessed as having either a positive or neutral impact on equity.

The Australian government has committed to developing a National Obesity Strategy [11]. This priority-setting study can be used to guide this strategy. Implementation of these 16 cost-effective interventions would require significant investment from the relevant federal and state government departments. Over the first three years, investment in all 16 interventions would require a budget of over AUD3 billion, with cost-savings over that timeframe limited to approximately AUD126 million, and additional downstream cost-savings being realised over the lifetime of the population. In 2014, Australia spent approximately AUD2 billion on prevention [56]. If one-tenth of this budget was spent on obesity prevention, the top five interventions on the league table could be implemented over the first three years (‘Alcohol price increase: uniform volumetric tax’, ‘Sugar-sweetened beverages tax -20%’, ‘Restricting television advertising of unhealthy foods’, ‘Package size cap on sugar-sweetened beverages’, and ‘Supermarket shelf tags on healthier products’). Budgetary impacts that may be important for decision-making but are not included in these evaluations relate to the significant revenue that can be achieved from taxation based interventions, with the 20% tax on sugar-sweetened beverages alone estimated to produce over AUD600 million in annual tax revenue [21]. Improved productivity in a healthier workforce and therefore increased income taxation revenue and lower welfare payments are additional benefits to the government that are not included in the analyses.

Implementation of interventions based solely on cost-effectiveness may not be politically feasible. Given that all the evaluated interventions are cost-effective, other aspects of interventions may be of importance in the prioritisation process. For example, if there is a political preference for interventions targeted at childhood obesity, there are several relevant dominant (‘Restricting television advertising of unhealthy foods’, ‘School-based intervention to reduce sedentary behaviour’ and 'School-based intervention to increase physical activity’) and cost-effective (‘Community- based interventions’) options. Alternatively, if high certainty of intervention effect on BMI outcomes is most important, there are two interventions that can be prioritised (‘Community- based interventions’ and ‘Financial incentives for weight loss provided by private health insurers’). This study highlights that effective government action will require co-ordinated action across state and federal governments and a whole-of-government approach with inter-departmental co-operation and co-ordination for successful implementation of the interventions.

The main strength of this study was that it used consistent methods to produce comparable evidence on the economic credentials of various obesity prevention strategies. The range of interventions evaluated included those that have been previously recommended by the WHO [8] and other health promotion bodies [57] as key components to an obesity prevention strategy (‘Sugar-sweetened beverages tax– 20%’, ‘Restricting television advertising of unhealthy foods’, and ‘National mass media campaign related to sugar-sweetened beverages’); new interventions that have not been previously evaluated from an obesity prevention perspective (‘Alcohol price increase: uniform volumetric tax price’, ‘Package size cap on sugar-sweetened beverages’, ‘Supermarket shelf tags on healthier products’, ‘School-based interventions to reduce sedentary behaviour’, ‘Restrictions on price promotions of sugar-sweetened beverages’, and ‘Workplace interventions to reduce sedentary behaviour’); interventions that have been implemented on a small scale or in other jurisdictions (‘Community–based interventions’ and ‘Workplace intervention to reduce sedentary behaviour’), and interventions that are currently being implemented by Australian governments (‘Menu kilojoule labelling on fast food’ and ‘Reformulation in response to the Health Star Rating (HSR) system’).

There are several limitations to this study. Firstly, despite efforts to maximise the comparability of interventions, the diversity of interventions meant that the evidence base for the modelling assumptions varied. We found that interventions with higher quality evidence were modelled using more conservative assumptions. To account for the uncertainty in the evidence base, the modelling of each intervention included extensive uncertainty and scenario analyses undertaken to provide more context when interpreting the results. These results have been reported in the publications of individual studies, however given that a single paper is unable to fully explain all the intervention scenarios evaluated (over 50 scenarios in total), we have only presented the base case ICERs in this paper. Secondly, this study was undertaken over six years, and at the outset, the 2010 base year represented the most current year available for all key data sources [32, 36, 58, 59]. While more contemporary data became available during the course of the study, the model was not updated to reflect these data. Thirdly, while it is well established that effective action on obesity is likely to require a suite of co-ordinated efforts, we only modelled the impact of single interventions implemented individually. This limitation relates to the availability of evidence, the structure of the model and the multi-sector focus of the interventions. Future research should focus on estimating the joint effects of multiple interventions to help inform decision-makers of the most cost-effective package of interventions. Fourthly, there was a lack of evidence of effectiveness of interventions in the area of agriculture, the built environment, environment and trade; limiting the sectors represented in the evaluations.

This study demonstrates the economic credentials of a suite of obesity prevention interventions relevant to the Australian setting. It provides the comparative evidence that can be used by governments to prioritise actions for obesity prevention. All 16 interventions evaluated were cost-effective and demonstrated great potential for long-term health benefits. Individual ranking of interventions will be improved with further research that explores the longer-term impacts of obesity prevention interventions and the joint impacts of several interventions implemented concurrently.

## Supporting information

S1 Table The Consolidated Health Economic Evaluation Reporting Standards (CHEERS) checklist. (DOCX)
