# Supplementary material for: Priority-setting for obesity prevention—The Assessing Cost-Effectiveness of obesity prevention policies in Australia (ACE-Obesity Policy) study
Source: PLoS One. 2020 Jun 19;15(6):e0234804. doi: 10.1371/journal.pone.0234804 (PMC7304600; doi:10.1371/journal.pone.0234804)
Supplement: S1 Table — (DOCX) [file pone.0234804.s001.docx]

**S1 Table: The Consolidated Health Economic Evaluation Reporting Standards (CHEERS) checklist**

| **Section/item** | **Item No** | **Recommendation** | **Section reported** |
| --- | --- | --- | --- |
| Title and abstract | | | |
| Title | 1 | Identify the study as an economic evaluation or use more specific terms such as “cost-effectiveness analysis”, and describe the interventions compared. | Title |
| Abstract | 2 | Provide a structured summary of objectives | Abstract |
| Introduction | | | |
| Background and objectives | 3 | Provide an explicit statement of the broader context for the study. | Introduction |
|  |  | Present the study question and its relevance for health policy or practice decisions. | Introduction: last paragraph |
| Methods | | | |
| Target population and subgroups | 4 | Describe characteristics of the base case population and subgroups analysed, including why they were chosen | Methods: *ACE-Obesity Policy model*; Table 2 |
| Setting and location | 5 | State relevant aspects of the system(s) in which the decision(s) need(s) to be made. | Methods: *Intervention selection* and *Overview of cost-effectiveness methods* |
| Study perspective | 6 | Describe the perspective of the study and relate this to the costs being evaluated. | Methods: *Intervention costs* |
| Comparators | 7 | Describe the interventions or strategies being compared and state why they were chosen. | Methods: *Intervention selection* and *Overview of cost-effectiveness methods;* Table 2 |
| Time horizon | 8 | State the time horizon(s) over which costs and consequences are being evaluated and say why appropriate. | Methods: *Impact of intervention on risk factors* and *The Ace-Obesity Policy model;* Table 2 |
| Discount rate | 9 | Report the choice of discount rate(s) used for costs and outcomes and say why appropriate. | Methods: *Cost-effectiveness modelling* |
| Choice of health outcomes | 10 | Describe what outcomes were used as the measure(s) of benefit in the evaluation and their relevance for the type of analysis performed. | Methods: *Overview of cost-effectiveness methods* and *The ACE-Obesity Policy model* |
| Measurement of effectiveness | 11a | Single study-based estimates: Describe fully the design features of the single effectiveness study and why the single study was a sufficient source of clinical effectiveness data. | Methods: *Impact of intervention on risk factors* and *Cost-effectiveness modelling* |
|  | 11b | Synthesis-based estimates: Describe fully the methods used for identification of included studies and synthesis of clinical effectiveness data. |  |
| Measurement and valuation of preference based outcomes | 12 | If applicable, describe the population and methods used to elicit preferences for outcomes. | Methods: *The ACE-Obesity Policy model* and *Cost-effectiveness modelling* |
| Estimating resource use and costs | 13a | Single study-based economic evaluation: Describe approaches used to estimate resource use associated with the alternative interventions. Describe primary or secondary research methods for valuing each resource item in terms of its unit cost. Describe any adjustments made to approximate to opportunity costs. | N/A |
|  | 13b | Model-based economic evaluation: Describe approaches and data sources used to estimate resource use associated with model health states. Describe primary or secondary research methods for valuing each resource item in terms of its unit cost. Describe any adjustments made to approximate to opportunity costs. | Methods: *Intervention costs* and *Cost-effectiveness modelling* |
| Currency, price date and conversion | 14 | Report the dates of the estimated resource quantities and unit costs. Describe methods for adjusting estimated unit costs to the year of reported costs if necessary. Describe methods for converting costs into a common currency base and the exchange rate. | Methods: *Intervention costs* |
| Choice of model | 15 | Describe and give reasons for the specific type of decision-analytical model used.  Providing a figure to show model structure is strongly recommended. | Methods: *The ACE-Obesity Policy model;* Figure 1 |
| Assumptions | 16 | Describe all structural or other assumptions underpinning the decision-analytical model. | Methods: *The ACE-Obesity Policy model* |
| Analytical methods | 17 | Describe all analytical methods supporting the evaluation. This could include methods for dealing with skewed, missing, or censored data; extrapolation methods; methods for pooling data; approaches to validate or make adjustments  (such as half cycle corrections) to a model; and methods for handling population heterogeneity and uncertainty. | Methods: *Cost-effectiveness modelling* |
| Results | | | |
| Study parameters | 18 | Report the values, ranges, references, and, if used, probability distributions for all parameters. Report reasons or sources for distributions used to represent uncertainty where appropriate. Providing a table to show the input values is strongly recommended. | Reported in individual studies. References in Table 2 |
| Incremental costs and outcomes | 19 | For each intervention, report mean values for the main categories of estimated costs and outcomes of interest, as well as mean differences between the  comparator groups. If applicable, report incremental cost-effectiveness ratios. | Table 3 |
| Characterising uncertainty | 20a | Single study-based economic evaluation: Describe the effects of sampling uncertainty for the estimated incremental cost and incremental effectiveness parameters, together with the impact of methodological assumptions (such as discount rate, study perspective). |  |
|  | 20b | Model-based economic evaluation: Describe the effects on the results of uncertainty for all input parameters, and uncertainty related to the structure of the model and assumptions. | Reported in individual studies. References in Table 2; uncertainty results in Table 3 |
| Characterising heterogeneity | 21 | If applicable, report differences in costs, outcomes, or cost-effectiveness that can be explained by variations between subgroups of patients with different baseline characteristics or other observed variability in effects that are not reducible by more information. | N/A |
| Discussion | | | |
| Study findings, limitations,  generalisability, and current  knowledge | 22 | Summarise key study findings and describe how they support the conclusions reached. Discuss limitations and the generalisability of the findings and how the findings fit with current knowledge. | Discussion |
| Other | | | |
| Source of funding |  | Describe how the study was funded and the role of the funder in the identification, design, conduct, and reporting of the analysis. Describe other nonmonetary sources of support. | Financial disclosure provided to journal |
| Conflicts of interest |  | Describe any potential for conflict of interest of study contributors in accordance with journal policy. In the absence of a journal policy, we recommend authors comply with International Committee of Medical Journal Editors recommendations. | The authors have declared that no competing interests exist. |
